# Angle stability and outflow in dual blade ab interno trabeculectomy with active versus passive chamber management

**DOI:** 10.1371/journal.pone.0177238

**Published:** 2017-05-09

**Authors:** Chao Wang, Yalong Dang, Susannah Waxman, Xiaobo Xia, Robert N. Weinreb, Nils A. Loewen

**Affiliations:** 1 Department of Ophthalmology, School of Medicine, University of Pittsburgh, Pittsburgh, Pennsylvania, United States of America; 2 Department of Ophthalmology, Xiangya Hospital, Central South University, Changsha, Hunan, China; 3 Shiley Eye Institute, Hamilton Glaucoma Center and Department of Ophthalmology, University of California San Diego, La Jolla, California, United States of America; Massachusetts Eye & Ear Infirmary, Harvard Medical School, UNITED STATES

## Abstract

**Purpose:**

To compare intraoperative angle stability and postoperative outflow of two ab interno trabeculectomy devices that excise the trabecular meshwork with or without active aspiration and irrigation. We hypothesized that anterior segment optical coherence tomography (AS-OCT) allows for a quantitative comparison of intraoperative angle stability in a microincisional glaucoma surgery (MIGS) pig eye training model.

**Methods:**

Twelve freshly enucleated porcine eyes were measured with AS-OCT at baseline, at the beginning of the procedure and at its conclusion to determine the anterior chamber depth (ACD) and the nasal angle *α* in degrees. The right and left eye of pairs were randomly assigned to an active dual blade goniectome (aDBG) and a passive dual blade goniectome (pDBG) group, respectively. The aDBG had irrigation and aspiration ports while the pDBG required surgery under viscoelastic. We performed the procedures using our MIGS training system with a standard, motorized ophthalmic operating microscope. We estimated outflow by obtaining canalograms with fluorescent spheres.

**Results:**

In aDBG, the nasal angle remained wide open during the procedure at above 90° and did not change towards the end (100±10%, p = 0.9). In contrast, in pDBG, ACD decreased by 51±19% to 21% below baseline (p<0.01) while the angle progressively narrowed by 40±12% (p<0.001). Canalograms showed a similar extent of access to the outflow tract with the aDBG and the pDBG (p = 0.513). The average increase for the aDBG in the superonasal and inferonasal quadrants was between 27 to 31% and for the pDBG between 15 to 18%.

**Conclusion:**

AS-OCT demonstrated that active irrigation and aspiration improved anterior chamber maintenance and ease of handling with the aDBG in this MIGS training model. The immediate postoperative outflow was equally good with both devices.

## Introduction

A range of ab interno, angle based procedures have become available to remove or bypass the resistance to conventional outflow in open angle glaucomas [1,2]. Although there is evidence that additional downstream elements exist [3], the trabecular meshwork (TM) is regarded as the principal pathology [4]. The difference amongst such microincisional glaucoma surgeries (MIGS) is the method of TM removal and how the anterior chamber is maintained. The removal methods include an active, plasma-mediated TM ablation [5], physical TM excision without [6] and with irrigation and aspiration [7,8], respectively, as well as TM bypass stents [9,10]. Until recently, the only system that maintained the anterior chamber actively with an irrigation and aspiration system was the Trabectome (Neomedix Inc.; Tustin, CA, United States), while the other surgical modalities used a viscoelastic device. Although these chamber maintaining techniques may seem interchangeable, surgeons who are new to MIGS have to move through a learning curve of five eyes to become safe but up to 30 eyes to become proficient even with active chamber management [11]. Viscoelastics are not recommended in plasma-mediated ablation because they can make visualization of the angle more difficult by trapping bubbles, debris, and blood and may cause carbonization of the ablation tip. Similar visualization challenges occur when viscoelastics are used with other TM excising MIGS that are made worse by a progressively shallowing anterior chamber and narrowing angle causing obscuration of the ablation target or billowing movements of the iris.

In this study, we sought to capture this behavior and analyze it. We hypothesized that anterior segment optical coherence tomography (AS-OCT) allows to quantify the intraoperative anterior chamber depth and angle opening. As detailed in the following, we compared a new dual blade goniectome with active aspiration and irrigation ports, to an existing, passive dual blade goniectome that requires viscoelastic to maintain the anterior chamber. We used our recently introduced MIGS training system to analyze the anterior chamber stability and outflow that can be achieved by an active [7,8] and a passive dual blade goniectome [6].

## Materials and methods

### Study design

**Fig 1:**
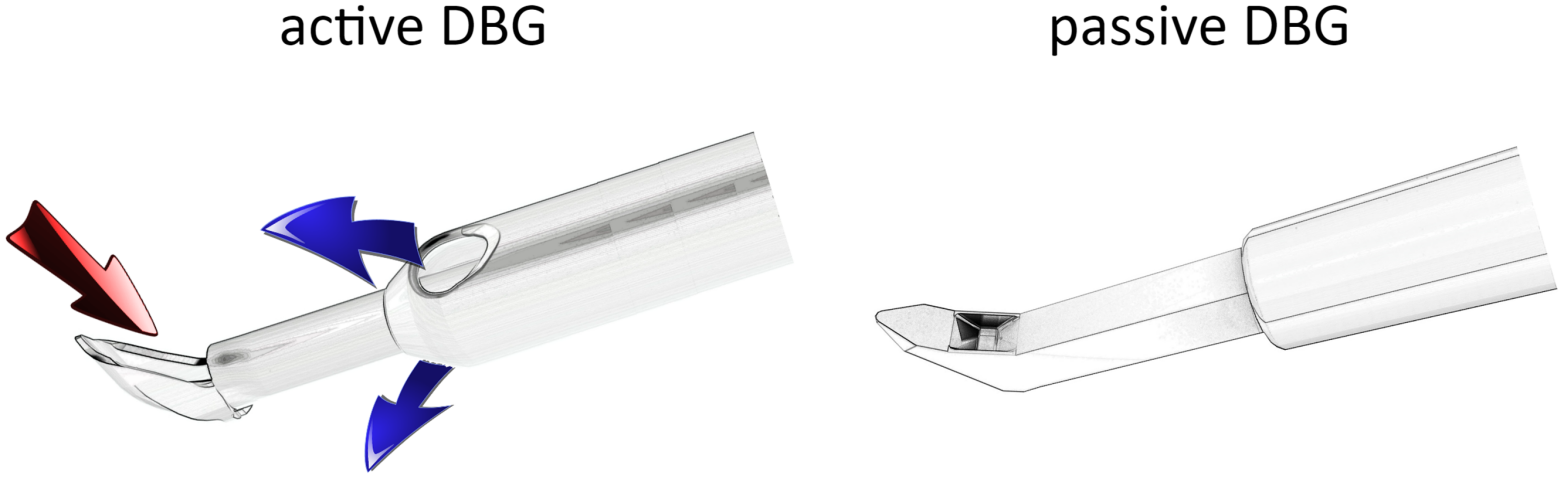
Active and passive dual blade goniectome. The active dual blade goniectome (active DBG, left [7,8]) has two irrigation ports that maintain the chamber (blue arrows). The trabecular meshwork is put under stretch by an angle shaped ramp and excised by a left and a right blade. The cut trabecular meshwork strip, blood and debris are aspirated into the tip (red arrow). The passive dual blade goniectome (passive DBG, right [6]) requires viscoelastic to maintain the anterior chamber. It also puts the trabecular meshwork under stretch by an angle shaped ramp and cuts the trabecular meshwork with a left and a right blade. The trabecular meshwork strip can be left in the eye or amputated and extracted with microforceps.

Twelve pig eyes were separated into equally paired groups of left and right eyes. This number was based on our prior studies [11–13] to maximize same-day comprehensive structure and function assays of eye pairs while providing a sufficient power to discover an outflow difference. They were prepared by removing excessive extraocular tissues and trimming the conjunctiva along the equator of the eyes. The eyes were assigned into two groups to undergo excisional (not plasma-ablative) ab interno trabeculectomy. The first group underwent surgery with a new active dual blade goniectome (“aDBG”; Neomedix Inc.; Tustin, California, United States), while the second group underwent surgery with a passive dual blade goniectome (“pDBG”; Kahook Dual Blade, New World Medical, Rancho Cucamonga, California, United States [6]), respectively. The design of the aDBG was similar to a plasma-ablative ab intero trabeculectomy instrument (Trabectome, Neomedix Inc.; Tustin, California, United States) and has a ramp that puts the trabecular meshwork (TM) under stretch before cutting it with a left and a right dual blade (Fig 1). The TM is aspirated by a central aspiration port at the level of the blades while the anterior chamber is maintained by two irrigation openings of the metal sleeve of the device. The aspiration and irrigation tubing can be connected to standard phacoemulsification machines, the peristaltic pump of the trabectome, a dedicated aDBG pump or through a simple gravity infusion in combination with an outflow tube that has a compressive clamp. The aspiration speed depends on what pump it is connected to while the irrigation is a function of aspiration and bottle height. The aspiration and irrigation behavior are equivalent to that of the trabectome if the peristaltic pump of that device is used. The pDBG has a ramp and dual blade that is similar to the first goniectome [7,8] but without aspirating or irrigating ports. This instrument requires a viscoelastic to maintain the anterior chamber.

We used one additional human donor rim (San Diego Eye Bank, San Diego, California, United States) to test the precision of excision and control of aspiration in a traditional upside-down wet lab holder. An inverted donor rim was secured with two 27-gauge needles and submerged in saline. The TM was engaged in a horizontal fashion, incised along the anterior and posterior lip of the TM for two clock hours going left while aspirating the emerging strip. Aspiration was stopped, and the instrument retracted to see whether the TM strip was intact before aspirating and releasing it again. The TM was then engaged with a pass towards the right starting at approximately two clock hours to the left of the prior excision’s end. The excision was stopped short of completion and the instrument retracted to inspect the TM strip. This experiment was explorative and its steps, in particular the re-aspiration and lack of strip amputation, were not meant to represent in vivo use.

The baseline anterior chamber depth (ACD) and nasal chamber angles (NCA) were acquired and measured by an anterior segment optical coherence tomography device (AS-OCT, Visante OCT, Carl Zeiss AG, Oberkochen, Germany) before perfusion with prewarmed Dulbecco's Modified Eagle's Media for 20 minutes (DMEM, Hyclone, GE Healthcare Life Sciences, Piscataway, NJ, USA) at a pressure of 15 mmHg. We then performed the assigned procedures with repeated imaging of ACD and NCA at the beginning and immediately at their conclusion. Outflow enhancement was evaluated by canalograms [14]. The changes of ACD, NCA and outflow increment, were used as the primary outcome evaluations. Use of human cadaver tissue was approved by the Institutional Review Board of the University of Pittsburgh and experiments were conducted in accordance with the tenets of the Declaration of Helsinki for the use of human tissue. No live vertebrate animals were used in this study. Pig eyes were obtained from a local abattoir. For this reason, no Institutional Animal Care and Use (IACUC) protocol was required.

### Preparation and pre-perfusion of the eyes

Twelve freshly enucleated porcine eyes were obtained from a local abattoir (Thoma Meat Market, Saxonburg, PA) within two hours of sacrifice. The eyes were identified as left or right based on adnexal materials that could include lids or nictitating membranes as well as muscles. Equally paired groups of left and right eyes were formed and assigned to the aDBG and pDBG groups. After carefully trimming the extraocular tissue and conjunctiva along the equator [12,13,15], the eyes were placed on cryogenic vial cups (CryoElite Cryogenic Vial #W985100, Wheaton Science Products, Millville, NJ) and a 30-gauge needle (REF 305128, BD PrecisionGlide^™^ Needles, BD Medical, Franklin Lakes, NJ) was inserted into the anterior chamber followed by perfusion with prewarmed DMEM.

### Anterior segment OCT

ACD and NCA were measured with AS-OCT at baseline using a 1310 nm wavelength. Eyes were placed in a holder, centered in the viewfinder and acquired as a centered, temporal to nasal (right eyes) or nasal to temporal (left eyes) scan. The same was also done at the beginning of the surgery and immediately at its conclusion. Structures in anterior segment scans were measured by aligning ruler and angle measurement tools to the appropriate structures. We were unable to use more recently introduced angle metrics such as the angle opening distance (AOD) from the scleral spur, the trabecular-iris space area (TISA) or the angle recess area (ARA) at 750 microns [16]. The scleral spur is not as prominent in porcine eyes as it is in humans [12] to be reliably detected on AS-OCT. Instead, we used the more traditional measures used for narrow angles, the anterior chamber angle in degrees (nasal angle opening *α*) and the central anterior chamber depth (ACD) in millimeters [17].

### Microsurgical model

The surgical steps were identical to that in human patients. The eyes were positioned under a surgical microscope (S4, Carl Zeiss Meditec, Jena, Germany) on a mannequin head mold with the temporal side towards the surgeon. All procedures were performed by a single surgeon (NAL) with comprehensive experience with both active and passive anterior chamber maintenance methods during ab interno trabeculectomies as used here. An iris-parallel, clear corneal incision of 1.8 mm was created with a keratome, about 2 mm anterior to the temporal limbus. The eye was rotated about 30° away from the surgeon while the microscope was tilted towards the surgeon. A goniolens (Goniolens ONT-L, #600010, Neomedix Inc., Tustin, CA, USA) was placed on the cornea to visualize the anterior chamber angle of the nasal side. The tip of the aDBG was inserted, and the anterior chamber deepened. The nasal TM was engaged with the tip pointing slightly up before straightening it out to a parallel position. A strip of trabecular meshwork was excised by holding the trabectome strictly parallel to the outer wall. The ab interno trabeculectomy continued counterclockwise for approximately 90°. The tip was then disengaged from the TM, rotated by 180° within the anterior chamber, and re-engaged at the original starting point. Ablation was then continued clockwise for a similar extent. After removal of the aDBG, the incision was sealed watertight with a drop of cyanoacrylate (KG585, Elmer Inc. Westerville, OH).

In the pDBG group, the anterior chamber was formed with a viscoelastic device (500 microliters, VISCOAT, Alcon Laboratories, Inc., Alcon, Fort Worth, Texas, United States), the eye was rotated away from the surgeon by 30° and the microscope tilted toward the surgeon by the same amount. The pDBG was carefully inserted into the middle of the anterior chamber, and the same goniolens as used with the aDBG (Goniolens ONT-L, #600010, Neomedix Inc., Tustin, CA, USA) was placed on the eye. The TM was engaged in the same fashion as described above, straightened out and passed along the outer wall over 90°. This was repeated in clock-wise fashion before the instrument was withdrawn and the incision sealed with cyanoacrylate.

### Canalograms

Enhancement of conventional aqueous outflow was estimated by measuring the extent to which fluorescent spheres were able to access the outflow tract after ab interno trabeculectomy as described [11,13] with minor modifications. Briefly, the corneal incisions were sealed with cyanoacrylate after TM ablation, the anterior chamber was cannulated with a 20-gauge hypodermic needle (150906, Exelint International, Redondo Beach, CA) and infused with carboxylated, yellow-green fluorescent 0.5 micron microspheres (100 times dilution in DMEM, F8813, Thermo Fisher, Waltham, MA) using a gravity perfusion system set to 15 mmHg. The aqueous outflow was visualized under a dissecting fluorescence microscope (SZX16, Olympus, Tokyo, Japan). The baseline frame was acquired as soon as the chamber was fully filled with FITC microsphere solution at a 680 x 510-pixel resolution with 8-bit depth and 2x2 binning at a 25 ms exposure while the end frame was taken at the 5 min after gravity perfusion. The raw fluorescence intensity was quantified by ImageJ (Version 1.50i, National Institute of Health). The outflow enhancement was measured by subtracting the baseline fluorescence intensity from the end frame by different quadrants.

### Statistics

Collected data was reported as the mean ± standard deviation unless stated differently. We used the *t*-test for same eye repeat testing of ACD and *α*. Fluorescence of groups was assessed using the ANOVA test. Differences were considered to be statistically significant for P>0.05. Correlation was tested with the Pearson test.

## Results

**Fig 2:**
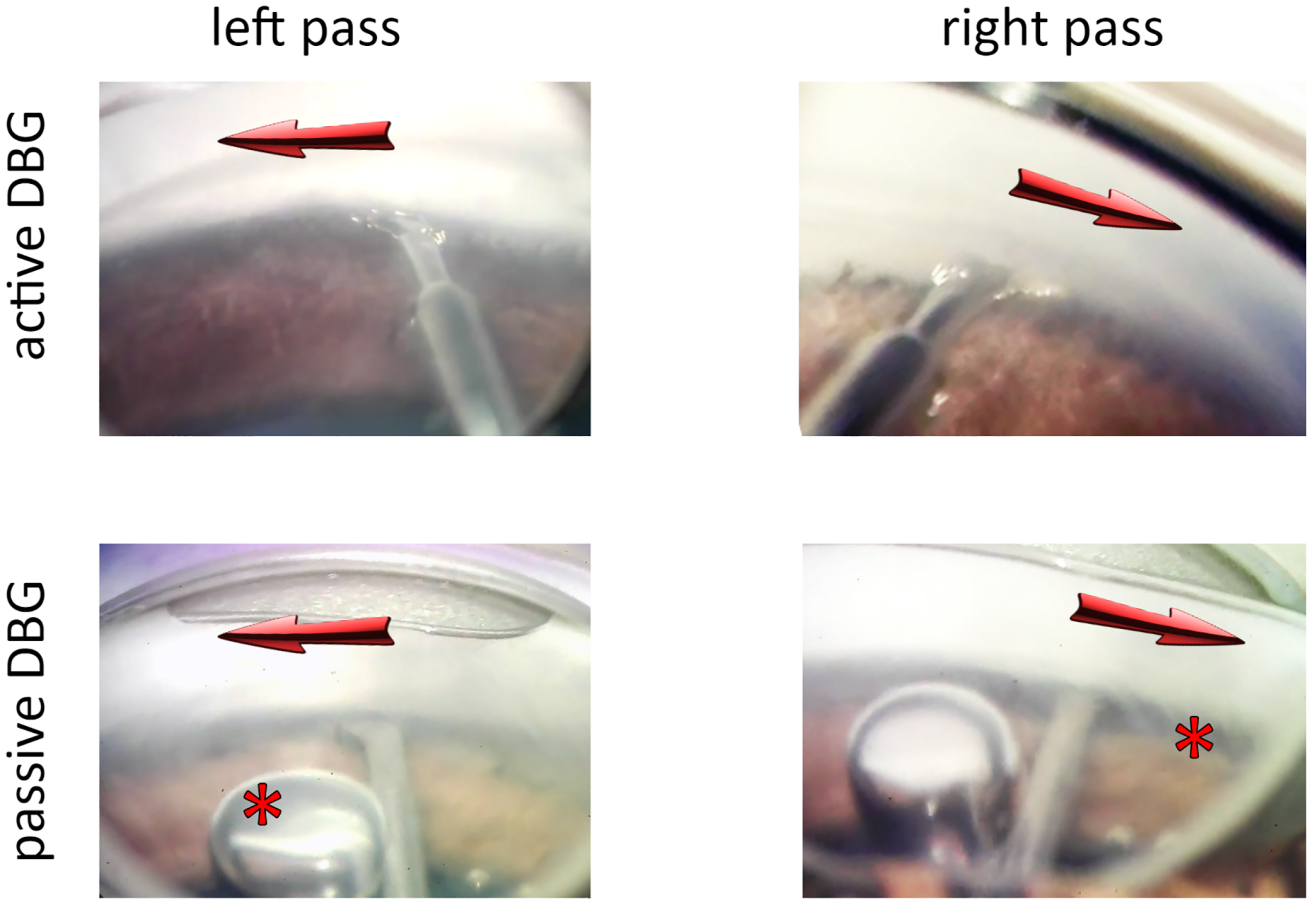
Intraoperative view of active and passive DBG. The active DBG (top) with an active irrigation and aspiration system. The anterior chamber was stable in both the left and the right pass. A supraphysiological deepening put the trabecular meshwork on tension and in direct view. Debris was aspirated. The passive DBG (bottom) required a viscoelastic to maintain the space. Air bubbles that were trapped in the viscoelastic (left asterisk) could not be removed without retracting the instrument from the eye. The anterior chamber became progressively more shallow as viscoelastic was displaced resulting in a narrowing angle and view obstruction from a billowing iris (right asterisk). Corneal striae from relative hypotony can be seen as well (right asterisk).

The entry into Schlemm’s canal in an inverted corneal rim was easy and allowed a drag free excision of the TM (S1 Movie). The TM fed into the aspiration opening of the tip as a single strip that could be retracted back out when the aspiration was stopped, and the attachment was not severed. The TM strip could also be re-aspirated and dislodged when desired. During intraocular surgery (Fig 2, **top**), the anterior chamber was maintained at a supraphysiological depth and placed a visible stretch on the TM as a result of a relative reverse pupillary block. This widened the angle and facilitated access to the TM. Similar to the corneal rim, the TM was easily excised and readily feed into the aspiration port in this species. This exposed the underlying white sclera of the outer wall of Schlemm’s canal like segments. Any debris could be readily aspirated, and visualization of the TM maintained for the entire surgery. The pressurization of the anterior chamber from the gravity fed infusion prevented corneal folds during gonioscopy. The fluid that egressed from the incision provided a saline fluid lake that maintained a bubble free interface between cornea and goniolens. The instrument was rotated without chamber collapse and the need to retract out of the eye. The fully pressurized anterior chamber made the often awkward backhand pass to the right straightforward. The aDBG had a sharp double edge which prevented drag of iris processes or TM during the entire procedure.

The ab interno trabeculectomy with the pDBG **(**Fig 2**, bottom)** was initially very similar to the aDBG while the viscoelastic provided a deep and stable anterior chamber. Viscoelastic injection often carried bubbles into the anterior chamber which were at times difficult to remove **(**Fig 2**, bottom left, red asterisk)**. When the anterior chamber was filled to the same depth of the aDBG, viscoelastic refluxed quickly. This was due to the lack of a tight seal between the viscoelastic cannula and the corneal incision, or the pDBG and the corneal incision, respectively. Engaging the TM was straightforward. The different tip design with more blunted blade sides and a more prominent ramp, when compared to the aDBG, caused noticeably more drag of iris processes and TM. Due to the lack of removal of material, the angle was harder to visualize and manipulation of the eye to counter this resulted in progressive loss of viscoelastic material with consecutive shallowing of the anterior chamber and narrowing of the angle. Rotation of the instrument gaped the incision resulting in further loss of viscoelastic, depressurization and corneal striae when the goniolens was placed. The backhand pass was more challenging and complicated by iris that billowed or wrapped around the tip and shaft of the pDBG **(**Fig 2, **bottom right, red asterisk)**.

**Fig 3:**
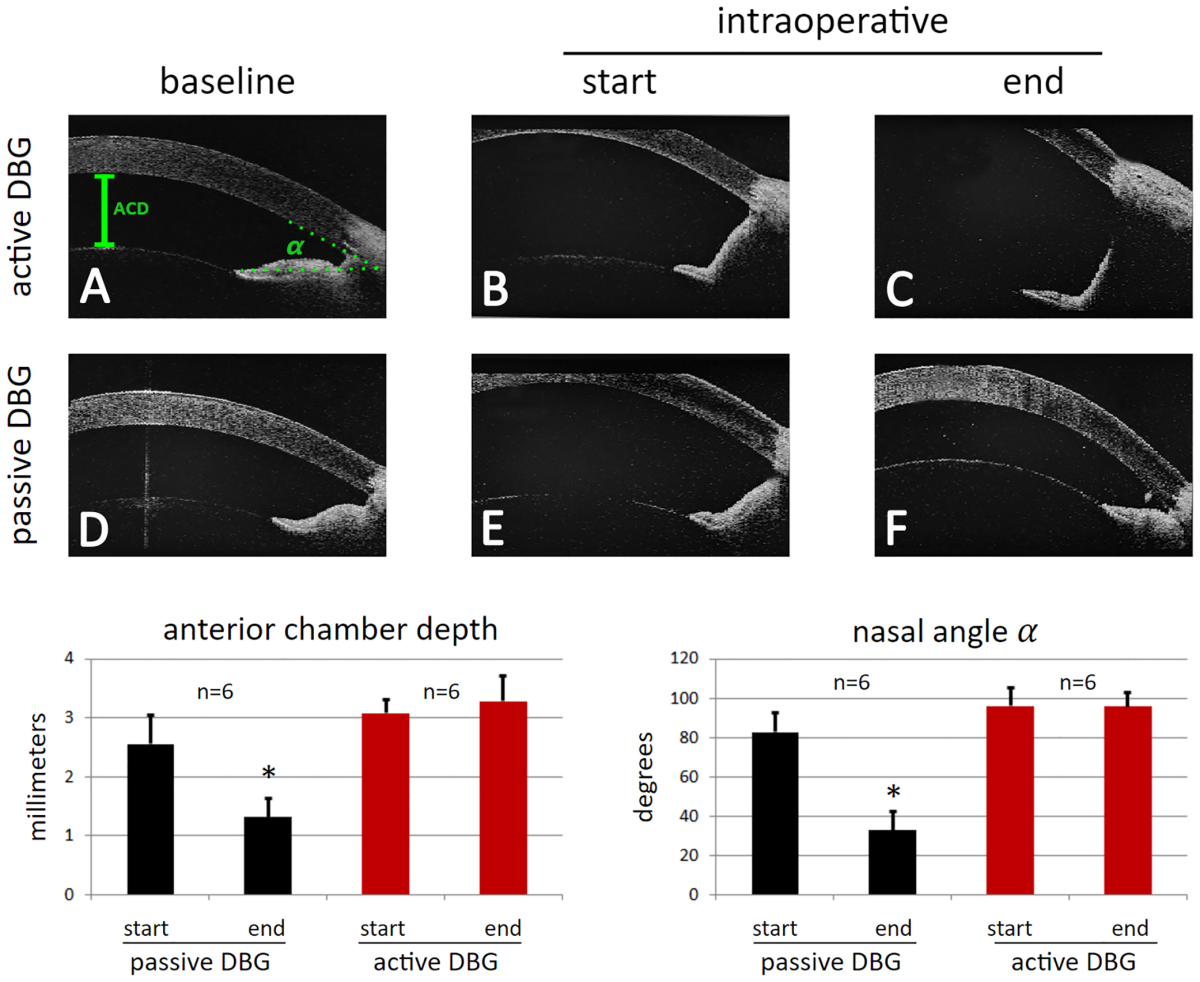
Intraoperative anterior segment optical coherence tomography (AS-OCT). Behavior and stability of the anterior chamber depth (ACD, green bar in A) and nasal angle opening (*α*, green dotted lines, A) was measured with AS-OCT. The irrigation and aspiration of the active dual blade goniectome maintains the space during surgery allowing unhindered view of the chamber angle (active DBG, B and C). The passive DBG utilizes a viscoelastic resulting in a progressively narrowing chamber angle (passive DBG, E and F). The anterior chamber depth decreased by 51±19% (p<0.01*) during passive DBG while it remained unchanged during active DBG (106±7%, p = 0.5). The anterior chamber angle decreased by 40±12% (p<0.001*) during passive DBG but it remained unchanged during active DBG (100±10%, p = 0.9).

We quantified the angle and anterior chamber changes with AS-OCT **(**Fig 3, **top, A-C)**. In aDBG, ACD increased from the baseline of 1.53±0.13 mm by 101% at the beginning and remained similar through the conclusion of the procedure (106±7% (p = 0.5)). The area of excised TM could be seen as a relatively signal sparse area between the sclera and the iris root (Fig 3C). The nasal angle remained wide open during the procedure at above 90° and did not change towards the end (100±10%, p = 0.9) (S1 Data).

In contrast, in pDBG, ACD increased from 1.68±0.13 mm by 52% after viscoelastic injection but decreased to below 21% of baseline at the conclusion. ACD shallowed during the pDBG procedure by 51±19% (p<0.01*, Fig 3D**)** and the angle progressively narrowed by 40±12% (p<0.001*, Fig 3F**)**. The AS-OCT of the example shown in Fig 3F shows how debris could occasionally remain possibly due to the challenging view towards the end of the procedure. In pDBG, the preoperative and postoperative ACD were strongly correlated with the degree of angle opening (r = 0.9 and 0.8, respectively) but the maximized ACD did not correlate in aDBG (S1 Data).

**Fig 4:**
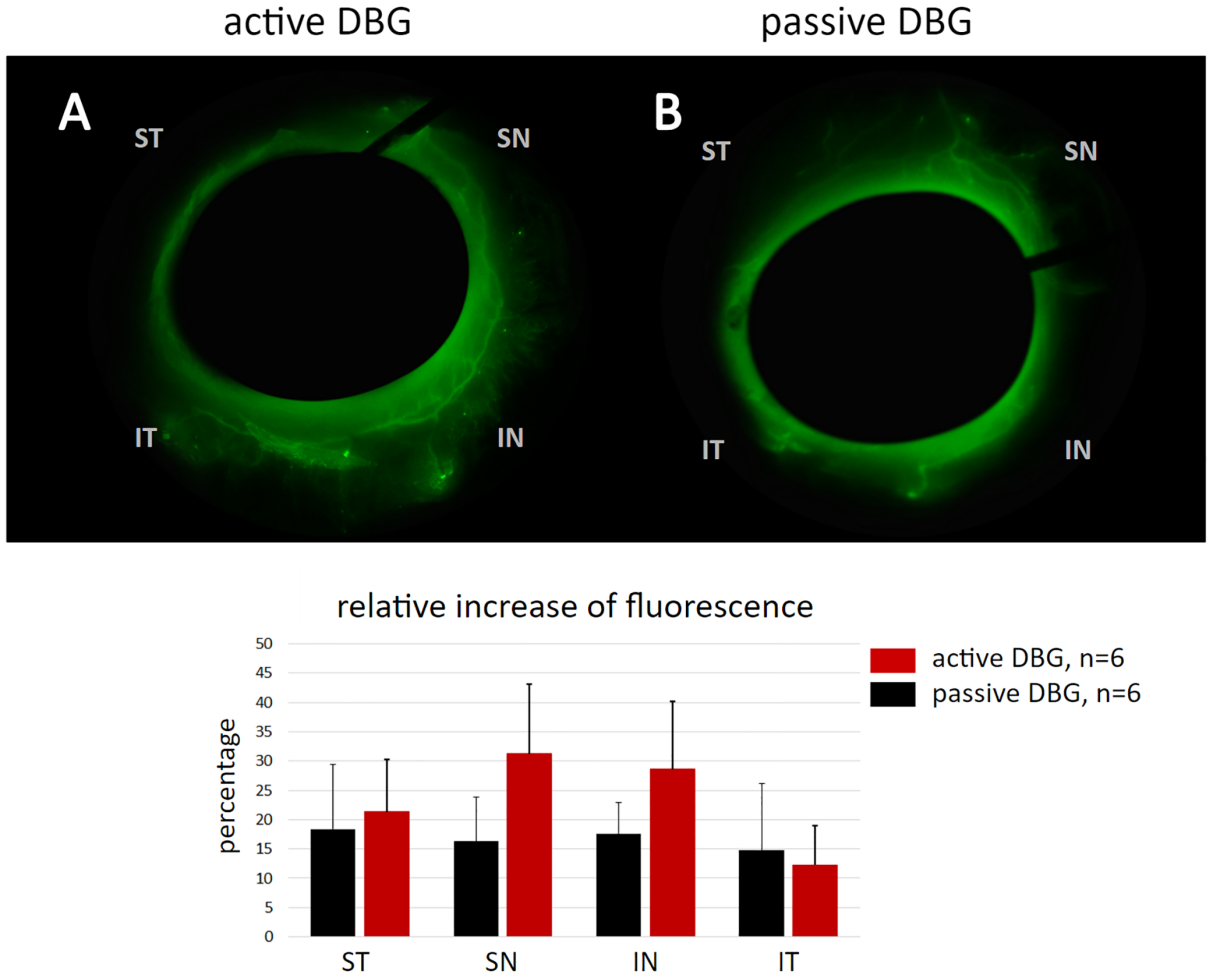
Outflow enhancement in aDBG and pDBG eyes. All quadrants experienced a significant increase of fluorescence (SN = superonasal, IN = inferonasal, IT = inferotemporal, ST = superotemporal quadrant). In average, there was more outflow enhancement in active dual blade gonioectome (aDBG) than in passive DBG eyes, but there was no statistically significant difference between six aDBG and six pDBG eyes examined here (p>0.05).

The microsphere canalograms highlighted sites where the TM had been removed and provided access to the downstream outflow system. A more than 180° filling of the superonasal and inferonasal most proximal outflow tract can be seen in Fig 4A with a canalogram of an aDBG eye that extended further when the circumferentially connecting channels of the outflow tract were accessed. Fig 4B shows a similarly extensive ablation with the pDBG and a circumferentially expanding outflow. The quantification of fluorescence changes indicated that the aDBG achieved the highest flow increase in the superonasal and inferonasal quadrants between 27 to 31% while the pDBG was relatively evenly changed by 15 to 18% **(**Fig 4, **bottom)**. There was no significant difference between aDBG and pDBG (p>0.05) in fluorescence increase observed (S1 Data).

## Discussion

In this study, we hypothesized that the intraoperative anterior chamber stability could be described and quantified with AS-OCT. We have comprehensive experience with an extensive range of angle surgical modalities both in training models [11,12] and in our patients [18–20]. As surgeon trainers, we are deeply familiar with the challenges of visualizing the target structure during surgery [1,2]. Traditional goniotomy, commonly used in pediatric glaucomas [21,22], can be performed without the need for a viscoelastic because the goniotome has a small, flat blade and a mostly self-sealing clear corneal incision [23]. Goniotomy and trabeculotomy techniques that do not remove the trabecular meshwork have historically been seen as less successful in adult glaucomas as anterior and posterior trabecular meshwork lips may reapproximate and occlude collector channel intakes. This is different from surgery in children where the elastic scleral spur retracts posteriorly after the incision separating the TM lips [24,25]. It was soon recognized that a TM stripping technique is more successful, but a modified anterior chamber access was necessary to do so without the use of intracameral viscoelastic [26]. We injected a commonly used, moderately dispersive viscoelastic [27] in the present study and used AS-OCT to image the angle changes during surgery. AS-OCT is the most established anterior segment imaging modality [17] and has the advantage of being able to image through structures that are reflective to the spectrum of visible wavelengths because it uses light at a wavelength of 1310 nm.

We chose two different ab interno trabeculectomy devices, the aDBG, and the pDBG, because of their similarity in TM excision that allows isolating the anterior chamber stability. The aDBG incorporates an irrigation and aspiration system that is identical to the one used in trabectome surgery. The tip design of both involves the ramp and tensioning of TM of the original goniectome [7,8]. Both are comparatively affordable and straightforward in design without the need for a high-frequency generator that ablates TM by molecularizing it with plasma and the overhead investment necessary. The aDBG can be connected to any irrigation and aspiration system, for instance to that of a phacoemulsification system.

As we experienced when operating on the human corneal rim, the aDBG allows gentle aspiration of a TM strip that is created. This may provide a useful tool to harvest TM for translational glaucoma studies as the material can be safely collected. The increase to a supraphysiological anterior chamber depth during the aDBG procedure is desirable because of the easy angle access this allows, even in narrow angles. This correlates to our clinical results of trabectome surgery in narrow angles [28] where a supraphysiological ACD allowed us to operate on angles previously thought to be contraindicated. The sclera of the pig is more elastic, and the iris-lens diaphragm is more displaceable than in human eyes. The intraocular experience in this model resembles the behavior of a moderately myopic eye. A viscoelastic principally has the same deepening effect as an active irrigation and aspiration, but the ACD and angle opening are progressively lost as the viscoelastic redistributes. In theory, this can be countered by reinjecting but is rarely done because of the reflux from the ablation site and mixing of blood from Schlemm’s canal with viscoelastic that results in further reduction of visibility.

The outflow studies presented here demonstrate at least equivalence in achieved outflow enhancement by the new aDBG and the established pDBG. While the averages of aDBG outflow increases appeared to be higher, there was no statistically significant difference. Based on the pilot data generated here we estimate that we would need at least 25 eyes to detect an outflow difference (unpaired *t*-test; alpha: 0.05, difference of mean: 14, expected standard deviation within groups: 17, desired power: 0.8).

It is encouraging that in this study the relatively poor visualization towards the end of the pDBG can be overcome by experience with angle surgeries, but this might not apply to surgeons new to them. We employed a microsphere-based canalogram in this study that represents a simplified, less time dependent method [11,13] compared to fluorescein canalograms that require careful timing but have a high temporal and spatial resolution [13,15]. In comparison, the fluorescent spheres indicate flow only after a direct connection to the downstream drainage system is obtained. Eyes without TM ablation show effectively no fluorescence in outflow channels as microspheres cannot bypass intact TM within the timeframe of this analysis [13]. Our outflow evaluation here demonstrates at least equivalence in achieved outflow enhancement by the new aDBG and the established pDBG.

The experiments presented here validate our training model for two additional techniques of ab interno trabeculectomy; it may be suitable for other ab interno glaucoma procedures that include suprachoroidal [29,30] and subconjunctival shunts [31].

Shortcomings of our study are that we did not use human donor eyes. Doing so would have led to a wide range of biological tissue properties unique to the donor and made the responsiveness of the iris-lens diaphragm less predictable due to the typically longer time from death to tissue availability and the more advanced age of the donor. The costs would have increased by 500 to 1000 fold. We also did not include a comparison of the aDBG and pDBG to the more established trabectome. Such plasma-mediated TM ablation is less traumatic because of the lack of sharp edges that may cut inadvertently into adjacent tissues. Compared to the aDBG and pDBG, the trabectome removes the TM over its entire width reliably and drag free; with this plasma-ablative device, there is less of a need to keep the tip perfectly centered in Schlemm’s canal. For new ab interno trabeculectomy surgeons, neither the aDBG nor the pDBG seems to offer compelling advantages over the existing trabectome but will probably allow avoiding upfront capital equipment expenses in most practice environments.

## Conclusion

In summary, we use a MIGS training system to assess in porcine eyes device performance, intraoperative anterior chamber depth and angle as well as the immediate postoperative outflow of two ab interno trabeculectomy instruments. Active irrigation and aspiration of the recently introduced aDBG allows high anterior chamber stability and visibility throughout surgery. The anterior chamber shallows and the angle narrows during surgery with the pDBG when using a viscoelastic with moderate cohesiveness. The immediate postoperative outflow enhancement is equally good with both devices.

## Supporting information

S1 Movie Trabecular ablation in an inverted corneal rim with the active dual blade goniectome. A human corneal rim was used to test the precision of excision and control of aspiration with the active dual blade goniectome (aDBG).
(MP4)

S1 Data Anterior chamber and fluorescence raw data. The raw data of 1) anterior chamber depth and nasal angle opening measured by AS-OCT, and 2) percentage increase of fluorescence intensity in the outflow tract as measured by microsphere canalograms.
(XLSX)
